# Outcomes of Disconnective Surgery in Intractable Pediatric Hemispheric and Subhemispheric Epilepsy

**DOI:** 10.1155/2012/527891

**Published:** 2012-02-09

**Authors:** Santhosh George Thomas, Ari George Chacko, Maya Mary Thomas, K. Srinivasa Babu, Paul Swamidhas Sudhakar Russell, Roy Thomas Daniel

**Affiliations:** ^1^Division of Neurosurgery, Department of Neurological Sciences, Christian Medical College, Vellore 632004, India; ^2^Division of Pediatric Neurology, Department of Neurological Sciences, Christian Medical College, Vellore 632004, India; ^3^Division of Neurophysiology, Department of Neurological Sciences, Christian Medical College, Vellore 632004, India; ^4^Department of Child and Adolescent Psychiatry, Christian Medical College, Vellore 632004, India; ^5^Division of Neurosurgery, Department of Clinical Neurosciences, CHUV, Rue du Bugnon 46, 1011 Lausanne, Switzerland

## Abstract

*Objectives*: To study the outcome of disconnective epilepsy surgery for intractable hemispheric and sub-hemispheric pediatric epilepsy. *Methods*: A retrospective analysis of the epilepsy surgery database was done in all children (age <18 years) who underwent a peri-insular hemispherotomy (PIH) or a peri-insular posterior quadrantectomy (PIPQ) from April 2000 to March 2011. All patients underwent a detailed pre surgical evaluation. Seizure outcome was assessed by the Engel's classification and cognitive skills by appropriate measures of intelligence that were repeated annually. *Results*: There were 34 patients in all. Epilepsy was due to Rasmussen's encephalitis (RE), Infantile hemiplegia seizure syndrome (IHSS), Hemimegalencephaly (HM), Sturge Weber syndrome (SWS) and due to post encephalitic sequelae (PES). Twenty seven (79.4%) patients underwent PIH and seven (20.6%) underwent PIPQ. The mean follow up was 30.5 months. At the last follow up, 31 (91.1%) were seizure free. The age of seizure onset and etiology of the disease causing epilepsy were predictors of a Class I seizure outcome. *Conclusions*: There is an excellent seizure outcome following disconnective epilepsy surgery for intractable hemispheric and subhemispheric pediatric epilepsy. An older age of seizure onset, RE, SWS and PES were good predictors of a Class I seizure outcome.

## 1. Introduction

Seizures are the most common neurological events of childhood with approximately 3–5% of children experiencing a seizure at some point in their lives 25% of whom subsequently go on to develop epilepsy [1]. The prevalence of epilepsy in India is 4.9 to 6.2 per 1000 population. Of these patients with epilepsy, 43% comprises of children and adolescents [2]. “Intractable epilepsy” is defined as a failure to respond to at least two antiepileptic drugs (AEDs) given over at least a two-year period [3]. A single definition for “intractable” epilepsy cannot suit all situations as definitions of intractability are individualized to the patient. Of these patients deemed to be intractable, approximately 50% are estimated to have surgically remediable epilepsy [4, 5]. “Hemispheric Epilepsy” (HE) refers to epileptiform activity in all four lobes of one hemisphere, and when it involves more than two lobes of the brain, it is termed “subhemispheric epilepsy” (SHE) [4, 6, 7]. These hemispheric brain lesions are commonly associated with early onset of catastrophic epilepsies and multiple seizure types that inhibit brain development. These respond well to early hemispheric/subhemispheric disconnective/resective surgeries [6–9].

## 2. Patients and Methods

An analysis was done of the pre- and postsurgical data of 34 children who underwent disconnective epilepsy surgeries, that is, a peri-insular hemispherotomy (PIH) or a peri-insular posterior quadrantectomy (PIPQ) for hemispheric/subhemispheric epilepsy from April 2000 to March 2011. The following were the selection criteria.

### 2.1. Selection Criteria for Peri-Insular Hemispherotomy [9, 13–15]

- Medical intractability.
- Contralateral hemiparesis with weakness of distal musculature of the upper and lower limbs.
- The hemisphere contralateral to the hemiparesis was shown by radiological (MRI/computerised tomography (CT)) and functional (scalp EEG/EEG video telemetry) imaging to have a unilateral diffuse abnormality and the remaining hemisphere was normal.

### 2.2. Selection Criteria for Peri-Insular Posterior Quadrantectomy [6, 7]

When the epileptogenic zone encompassed large areas of the temporal, parietal and occipital lobes with sparing of the frontal lobe, a PIPQ was performed. The decision was dependant on good concordance between the imaging (MRI, CT, nuclear studies), EEG, clinical and neuropsychological evaluations, and a clear localization of the lesion to the unilateral-affected region. The indications for PIPQ were the same as for hemispheric epilepsy, the pathology being localized to involve the temporal, parietal, and occipital lobes. The presence of residual voluntary motor function of the contralateral distal musculature, that is, finger opposition and foot tapping, was the indication for PIPQ preserving eloquent uninvolved sensorimotor cortex.

### 2.3. Presurgical Evaluation

All patients went through a presurgical evaluation including a study of the seizure semiology, neurological examination, multiple electroencephalogram (EEG) examinations (video telemetry), magnetic resonance imaging (MRI), and neuropsychological evaluation. The focus of the evaluation was to identify a surgically remediable epilepsy syndrome with good electro-clinico-radiological concordance. If the structural lesion responsible for epilepsy could be safely removed without causing deterioration in the functional status, the patient was considered for epilepsy surgery. However, if there was no concordance in investigations in the Phase I evaluation, the patient would enter a Phase II evaluation which would include prolonged invasive EEG monitoring, nuclear medicine studies (positron emission tomography (PET) and single photon emission computerized tomography (SPECT)), and a WADA (Sodium amylobarbital) test. However, none of our patients required a phase II evaluation.

### 2.4. Surgery

**Figure 1:**
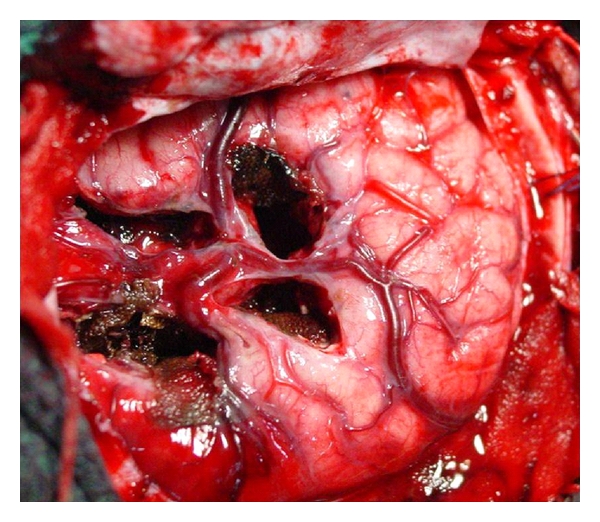
Intraoperative photograph of peri-insular hemispherotomy (PIH) showing the supra- and infrainsular windows.

A PIH was performed in all cases with hemispheric epilepsy. (Figure 1) This is a surgical method of functional hemispherectomy that enables disconnection of the hemisphere through peri-insular windows requiring limited removal of the fronto-parieto-temporal opercular cortices. Following the surgical principles of “anatomical subtotal removal of the hemisphere and complete disconnection,” the PIH is a radical hemispheric tractotomy based on the concept of maximum disconnection with minimal excision. It resulted from the demonstration that the hemisphere could be disconnected, made nonfunctional, through very small removal of brain tissue [9, 14, 15].

**Figure 2:**
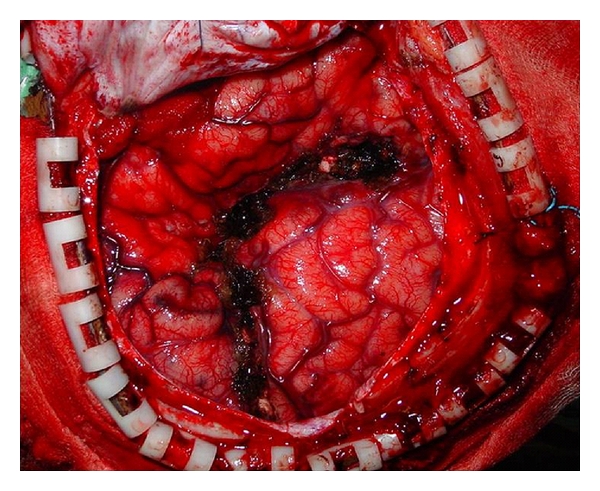
Intraoperative photograph of peri-insular posterior quadrantectomy (PIPQ) showing the line of disconnection.

A PIPQ was performed in all cases of subhemispheric epilepsy and the surgery tailored to encompass the whole epileptogenic lesion yet preserving the central region, which is still functional. (Figure 2) In this technical variant, there is a minimal removal of brain tissue but complete disconnection of the remaining major part of the abnormal cortex, which is left anatomically intact and viable by preservation of the arteries and veins irrigating these lobes. The primary motor and sensory cortices are identified and recognized from the study of the MRI and correlation with intraoperative surface anatomy, based on gyral pattern, arteries, and veins [6, 7]. The identification of the functional cortex is also aided by electrophysiological means under general anaesthesia before the disconnection. This identification maximizes both the extent of resection and the safety of surgery [6, 7]. In this variant, the mesial temporal structures are resected, but the temporal neocortex is disconnected and not resected followed by a parieto-occipital disconnection [6, 7].

### 2.5. Statistical Analysis

The data was analyzed with nonparametric tests because of the small sample size among the children with poor outcome following surgery (Engel's class II–IV). Nonparametric *χ* ^2^ square test and Mann-Whitney *U* test was were to compare the categorical and continuous variables between the groups. *P* < 0.05 (two tailed) was considered significant and data was analyzed using SPSS (version 19).

## 3. Results

### 3.1. Demography

**Figure 3:**
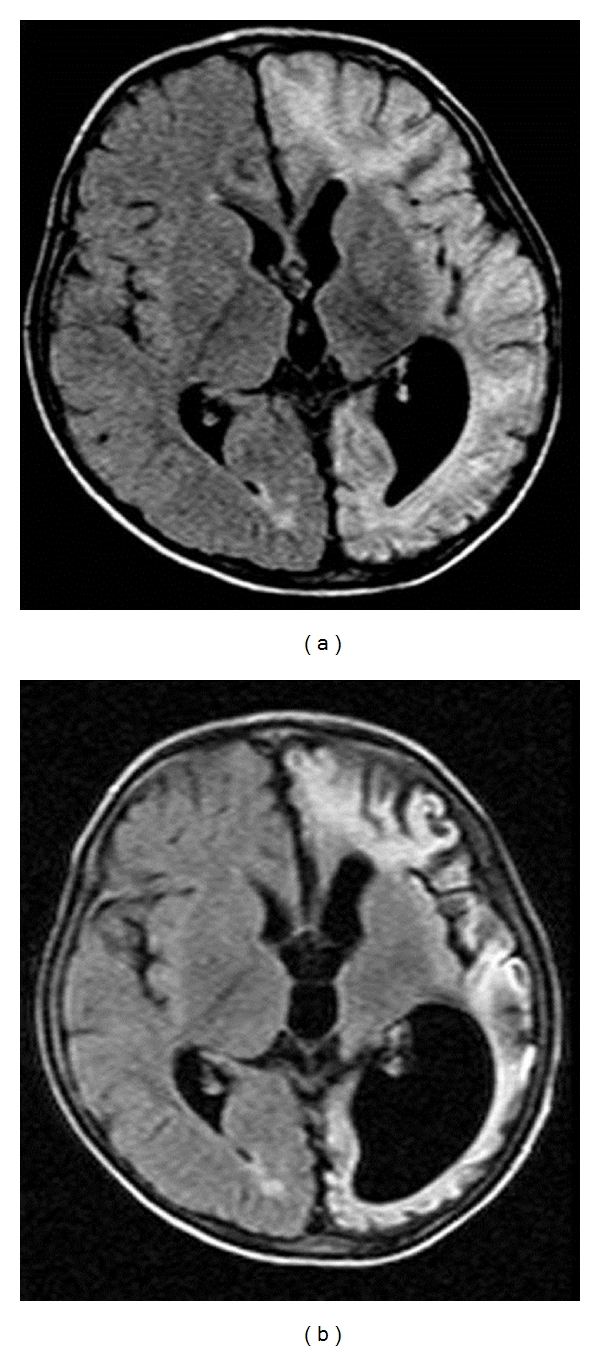
T2W Axial flair MRI images of a patient with Rasmussen's encephlitis taken six months apart showing progression of disease.

**Figure 4:**
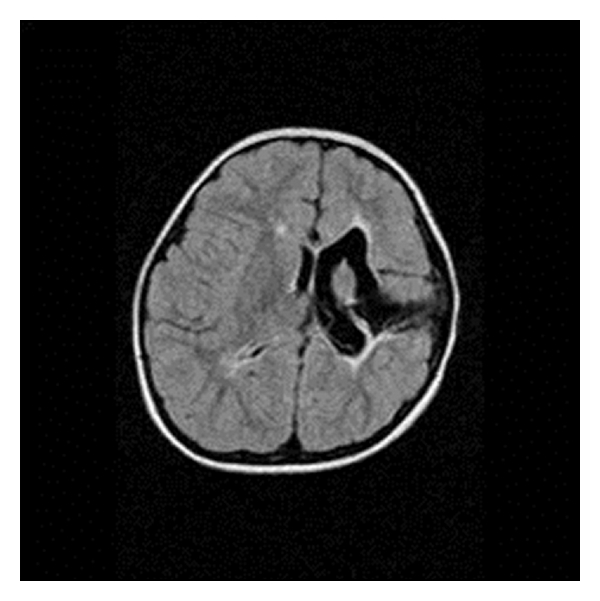
T2W Axial flair MRI image of a 3-year-old boy with infantile hemiplegia seizure syndrome (IHSS).

**Figure 5:**
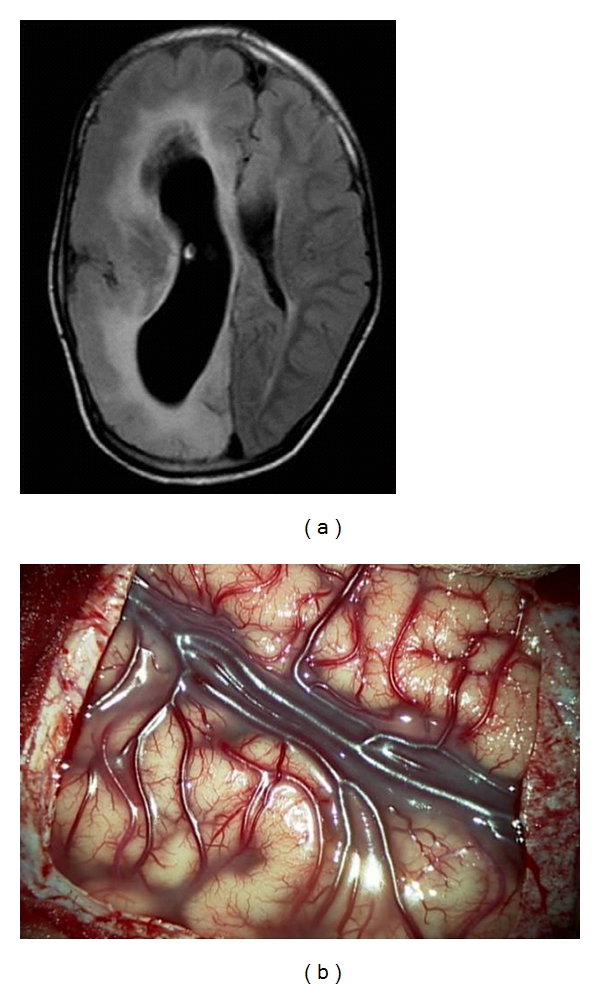
(a) T2W Axial Flair MRI Image of a patient with right hemimegalencephaly showing an enlarged right hemisphere with thickened cortical mantle, distorted ventricular anatomy, and widened gyri, (b) intraoperative photograph.

**Figure 6:**
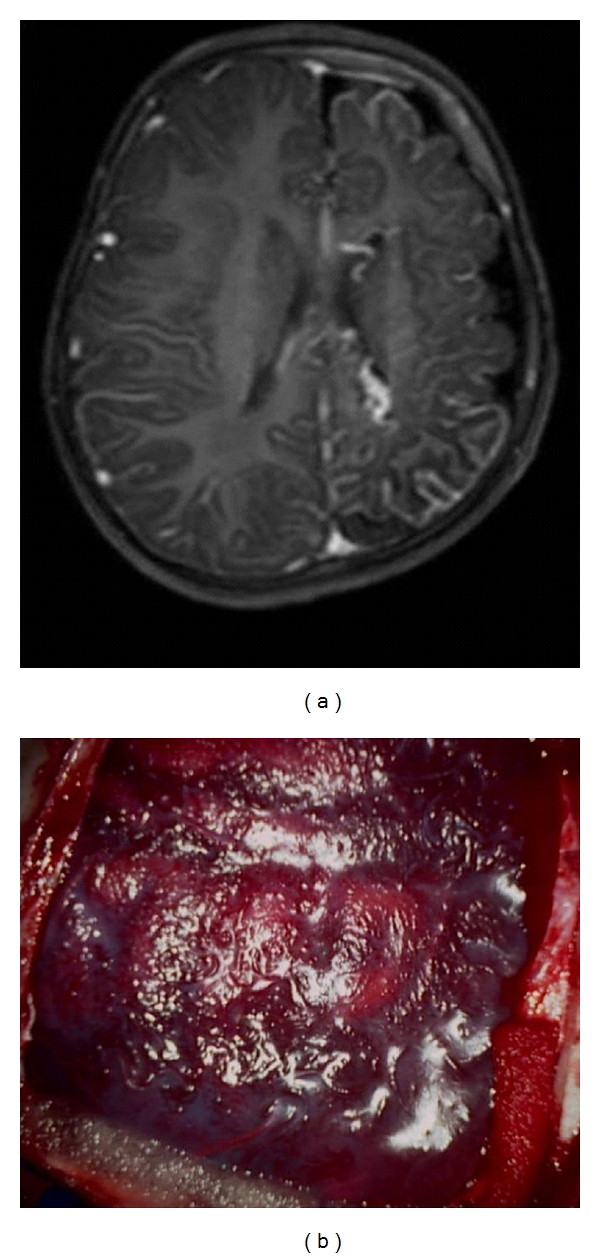
(a) T1W Axial gadolinium MRI image of a patient with Sturge Weber disease showing left hemispheric leptomeningeal angiomatosis, (b) intraoperative photograph.

**Figure 7:**
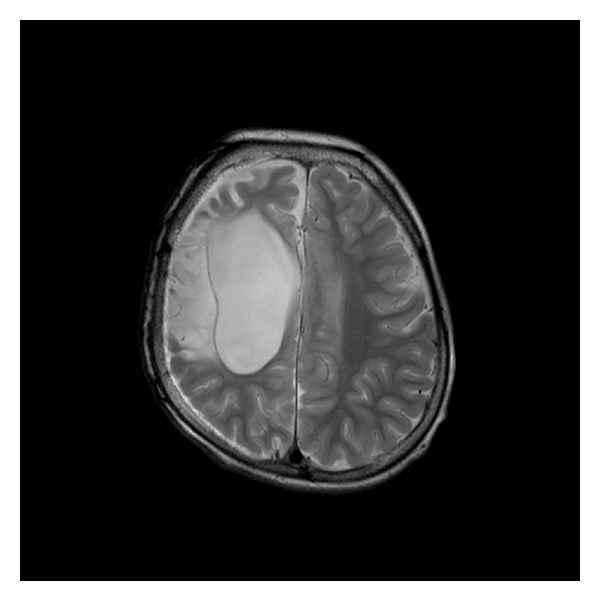
T2W Axial MRI image of a 5-year-old boy who presented with intractable epilepsy secondary to postencephalitic sequelae (PES) causing damage to the right side.

There were 34 children with a mean age of 7.9 years. There were 22 (64.7%) males and 12 (35.3%) females. The mean age of seizure onset was 3.8 years (range: from neonates to 12 years). The mean duration of epilepsy was 4 years (range: from 3 months to 14 years). 28 children (82.3%) had a seizure frequency of ≥2 episodes/day, while 11 (32.3%) had at least one episode of status epilepticus prior to surgery. Twenty-two of them (64.7%) were on more than two AEDs. Epilepsy was due to Rasmussen's encephalitis (RE; *n* = 11) (Figure 3), infantile hemiplegia seizure syndrome (IHSS; *n* = 12) (Figure 4), hemimegalencephaly (HM; *n* = 3) (Figure 5), Sturge Weber syndrome (SWS; *n* = 4) (Figure 6), and postencephalitic sequelae (PES; *n* = 4) (Figure 7). Twenty-seven (79.4%) patients underwent PIH for lesions causing hemispheric epilepsy, and seven (20.6%) underwent PIPQ for lesions causing subhemispheric epilepsy. The mean followup was 30.5 months.

### 3.2. Seizure Outcome and Their Predictors

The seizure outcome after surgery was assessed by Engel's classification [4] and is described along with followup in Table 1. We recorded 11 variables and analyzed them to arrive at predictive variables of a complete seizure freedom. (Tables 2 and 3). We found that age of seizure onset (*P* = 0.03) and the etiology of the disease causing epilepsy (*P* = 0.007) were predictive variables for the same. An older age of seizure onset pointed to good seizure outcome following surgery. Patients with HM had the worst seizure outcome. Patients who had RE, SWS, PES, and IHSS had good seizure outcomes following surgery.

### 3.3. Cognitive Outcome and Their Predictors

The results of our cognitive outcomes that have been published before [12] show that the mental and social age showed a steady increase after surgery. However, in the long term, intelligence quotient (IQ) showed only a gradual gain on followup. Older age of onset of seizures and a shorter duration of seizures prior to surgery were predictive of positive cognitive gains following surgery [12].

### 3.4. Complications

Complications in this series included pseudomeningocele in 2 (5.8%) and low pressure hydrocephalus in 1 (2.9%) who required a low pressure ventriculoperitoneal shunt. This is quite low as compared to other published series (Table 4) [9–11]. We also had 15 patients who developed postoperative fever, but CSF cultures were sterile. This could have been due to an aseptic meningitis due to the presence of blood products in the disconnected cavity.

## 4. Discussion

### 4.1. Seizure Outcome

Intractable epilepsy due to hemispheric/subhemispheric epilepsy respond well to PIH or PIPQ. Our series documents 91.1% seizure freedom, which is similar to other published studies [9, 12, 16–21]. Following functional hemispherectomy, seizure free outcomes have ranged from 52 to 90% [9, 16–21]. This range could be explained by differences in patient selection or technical pitfalls such as incomplete removal in anatomical hemispherectomy and incomplete disconnection in functional hemispherotomy [14, 19]. We found the age of seizure onset to be a strong predictor of seizure freedom (Table 2). This finding is also supported by other studies [3, 22, 23]. A younger age of seizure onset is usually seen in children with developmental cortical malformations and multifocal epilepsy, which are proven to have poor seizure control [3].

The etiology of the disease producing epilepsy is also a strong predictor of seizure freedom (Table 3). This has been shown in other studies as well [12, 18–22]. The best results in our series were obtained in children suffering from RE, SWS, PES, and IHSS (Table 3). IHSS and SWS are seen in the perinatal age group, and they are generally unilateral with complete sparing of the opposite side. There has been evidence to support that in such cases, early surgical intervention facilitates good seizure control and helps the uninvolved hemisphere to develop and take over the functions of both sides [4]. However, in one patient with IHSS, we had a Class II seizure outcome and this was in the initial part of the series, which could be attributed to incomplete disconnection, and this patient was lost to follow up. All patients with RE had a Class I seizure outcome as this disease also strictly affects only one hemisphere. However, the seizure outcome in patients with HM was not satisfactory as described in literature, and this has been reported in other series too [9, 17–21]. The likely explanation could be the presence of migrational abnormalities in the so-called “preserved hemisphere” or early development of an epileptic encephalopathy, already in utero [9].

### 4.2. Social and Adaptive Outcome

Our data with respect to cognitive outcome has been published earlier [12]. We found a significant gain in the mental and social age in the immediate postsurgical period which continued into the second and third years of followup. Our study [12] also showed that older age of onset of seizures showed positive mental and social age gains at followup. This finding is also supported by other studies [3, 24]. Onset of intractable epilepsy within the first 24 months of life is a significant risk factor for mental retardation, especially if seizures occur daily [12, 24]. Most of these children have a low DQ/IQ to begin with and the improvement in cognitive skills after surgery is poorer in these children. Furthermore, our study [12] also showed that a shorter duration of seizures prior to surgery is predictive of positive mental and social age gains, a finding that is in keeping with those of Freitag and Tuxhorn [22] and Basheer et al. [21]. In general, children with IE in our country are not exposed adequately to environmental stimuli as schooling is discontinued in the presence of seizures [12]. With good postoperative seizure control, the child's attention capacities increase and they engage in social interaction with their family and peers. An improved school attendance due to seizure freedom improves their adaptive skills that in turn helps the child improve his performance in activities of daily living [12].

## 5. Conclusion

In our experience, over 90% of children with hemispheric and subhemispheric epilepsy syndromes achieve an excellent seizure outcome with less morbidity following epilepsy surgery. Age of seizure onset and etiology of the disease causing epilepsy are independent predictive variables of a good seizure outcome. Following seizure freedom, improvement of function in the residual brain occurs that in turn leads to improvement in adaptive and social functions and quality of life.

## Figures and Tables

**Table 1:** Postoperative seizure outcome with followup.

Engels	Description	*N* = 34 (100%)	Mean followup (months)
Class I	Complete seizure freedom	31 (91.1%)	32.3
Class II	>90% reduction in seizures	2 (5.8%)	18
Class III	75–90% reduction in seizures	—	—
Class IV	No improvement or worsening after surgery	1 (3.1%)	24

**Table 2:** Presurgery predictors of age, seizures, and cognition on postsurgery seizure outcome.

Variables	Engels Class I (*n* = 31)		Engels Class II–IV (*n* = 3)		Statistics *Z* value	*P* Value
	Mean	SD	Mean	SD		
Age at the time of surgery	8.10	4.18	6.43	7.64	−0.76	0.44
Age of seizure onset	4.078	3.84	0.07	0.03	−2.15	0.03
Frequency of seizures/day	113.33	269.29	6.50	2.12	−0.17	0.86
Total duration of seizures before surgery	4.11	3.39	2.05	2.75	−1.12	0.26
IQ/DQ (pre op)	59.20	17.97	35.00	0.01	−1.41	0.15

**Table 3:** Presurgery predictors of gender, disease, and type of surgery on postsurgery seizure outcome.

Variables		Engels Class I (*n* = 31)		Engels Class II–IV (*n* = 3)		Statistics *χ* ^2^	*P* Value
		*n*	%	*n*	%		
Gender	Male	20	64.5%	2	66.7%	0.006	0.94
	Female	11	35.5%	1	33.3%		
	RE	11	35.4%	0	0%		
	IHSS	11	35.4%	1	33%		
Disease	SWS	4	12.9%	0	0%	14.23	0.007
	HM	1	3.2%	2	66.7%		
	PES	4	12.9%	0	0%		
Side	Right	14	45.2%	2	66.7%	0.50	0.46
	Left	17	54.8%	1	33.3%		
Status epilepticus (pre-op)	Yes No	10 17	37% 63%	1 1	50% 50%	0.13	0.71
Epilepsia partialis continua (pre-op)	Yes	6	22.2%	0	0%	0.56	0.45
	No	21	77.8%	2	100%		
Type of surgery	PIH	25	80.6%	3	100%	0.70	0.40
	PIPQ	6	19.4%	0	0%		

**Table 4:** Comparison of surgical techniques, seizure outcomes, and complications.

Author and year	Technique	No. of patients	Engels Class1 outcome	Complications	Mean followup (years)
Schramm et al. [10]	Transylvian hemispherectomy	20	88%	Mortality 5% Infection 5%	3.8
Villemure and Daniel [9]	Peri-insular hemispherotomy	43	90%	Mortality 2% Hydrocephalus 2% Hemorrhage 5%	9
Delalande et al. [11]	Vertical hemispherotomy	83	74%	Mortality 4% Hydrocephalus 16%	4.4
Thomas et al.; our series	Peri-insular hemispherotomy and posterior quadrantectomy	34	91.1%	Mortality 0% Hydrocephalus 2.9% Pseudomeningocele 5.8%	2.5
